# Exogenous Spermidine Promotes Germination of Aged Sorghum Seeds by Mediating Sugar Metabolism

**DOI:** 10.3390/plants11212853

**Published:** 2022-10-26

**Authors:** Min Zhang, Bang Li, Zuliang Wan, Xiaofei Chen, Chang Liu, Chunjuan Liu, Yufei Zhou

**Affiliations:** 1 College of Agronomy, Shenyang Agricultural University, Shenyang 110866, China; 2 Crop Research Institute, Anhui Academy of Agricultural Sciences, Hefei 230031, China

**Keywords:** aged sorghum seeds, germination, spermidine, starch, sugar metabolism

## Abstract

Starch, a substance stored in seeds, is the main source of energy for germination in sorghum seeds. However, as the seeds age, the catabolism of seed starch is affected, thereby seriously damaging germination ability. In this study, we aimed to understand how exogenous spermidine promoted germination in aged sorghum seed. Our phenotypic analysis indicated that exogenous spermidine not only significantly improved the germination rate, germination potential, germination index, and vigor index of aged seeds, but also increased the root and shoot length after germination. Further, physiological analysis showed that exogenous spermidine increased the content of soluble sugar by upregulating the activity of amylase and sucrose invertase. Exogenous spermidine also improved the activities of key enzymes in glycolysis, the tricarboxylic acid cycle, and the pentose phosphate pathway of aged sorghum seeds. Interestingly, exogenous spermidine protected the mitochondrial structure of aged seeds, which was consistent with the increase in the respiration rate and ATP content during seed germination. Moreover, qRT-PCR analysis revealed that exogenous spermidine induced the expression of key genes related to starch and sugar metabolism in aged sorghum seeds. In conclusion, our study demonstrated that exogenous spermidine promoted aged sorghum seed germination by regulating starch and sugar metabolism.

## 1. Introduction

Seed aging becomes inevitable due to unfavorable storage conditions and prolonged storage [1,2,3]. As the seed ages, it undergoes physiological degradation, including inhibition of root and shoot growth, affecting seed germination and, consequently, resulting in poor crop growth [2,4,5,6]. Previous studies have primarily focused on examining the effects of seed aging on seed germination capacity of corn, rice, oats, tobacco, and wheat [5,7,8,9,10]. However, the physiological mechanism of poor germination of crop seeds due to aging remains unclear. Sorghum (*Sorghum bicolor* L. Moench) is mainly planted in arid and semiarid areas of Africa and Asia, and is considered a multipurpose crop for food, brewing, and biofuel, etc. [11,12,13]. Therefore, further studies are needed to investigate the specific cause of germination deterioration and to explore effective alleviating approaches, since sorghum is susceptible to aging [14].

Seed germination is a physiological activity that is regulated by endogenous and exogenous factors [15,16]. This complex physiological process involves activities such as water absorption, storage degradation, seed respiration, transcription product (mRNA) synthesis, and mitochondrial repair and proliferation, all of which require an adequate energy supply [15,17,18]. The energy supply for seed germination and emergence comes from the catabolism of stored substances in the seed before the seedlings acquire photosynthetic capacity [19,20,21]. The mobilization of internal storage reserves during seed germination varies with the amount and type of storage substances. Starch is an important storage material in sorghum seeds, with a general content of more than 70% of total seed weight [22,23]. Noticeably, the degree of starch decomposition is the highest during germination [24,25]. Starch is an important source of energy and is closely related to seed vigor and the germination process. Low rates of starch metabolism at the germination stage causes failure of seed germination [26,27]. Soluble sugar produced by starch metabolism is the main substrate of energy produced by respiration. A few studies have noticed a positive correlation among the content of soluble sugar, amylase activity, and the rate of seed germination [28,29]. Sucrose is the primary transport sugar in plants that is mobilized to grow leaf tissue, twigs, or roots. Sucrose must be metabolized into soluble sugar, such as glucose and fructose, in these growing and developing tissues before it can be used [27]. The mobilization of starch in seed germination is a key factor affecting seed germination. Whether the low germination rate of aging seeds is related to inhibition of starch transformation ability during seed germination is unclear and needs further investigation.

The soluble sugar produced by starch decomposition is eventually catabolized into ATP—the most direct form of energy for seed germination and growth. ATP levels directly affect the germination ability of seeds. Earlier studies have reported that exogenous ATP inhibits the *BoSnRK2* pathway by maintaining a high-level energy state, and further promotes the level of nutrition and antioxidants [30]. Further, ATP production through soluble sugar involves the important sugar metabolism pathway. Sugar metabolism mainly includes glycolysis (EMP), the tricarboxylic acid cycle (TCA), and the pentose phosphate pathway (PPP). Previous research on rice indicates that the respiration rate and energy levels during early germination are highly dependent on the EMP pathway, as it breaks down glucose to produce pyruvate and energy under aerobic conditions [31,32]. The TCA involves the oxidative phosphorylation of pyruvate to produce ATP, and key TCA cycle enzymes are upregulated after seed germination [33]. Further, PPP also participates in glucose metabolism and contributes to seed germination by providing intermediate materials for seed germination [34,35]. Mitochondria participate in energy metabolism, and thus facilitate cell growth. Noticeably, energy metabolism in mitochondria begins rapidly with the progression of seed germination [36]. An earlier transmission electron microscopy study on seeds reported that mitochondria had a low matrix density, discontinuous outer membrane, and lack of cristae, which resulted in a low capacity of ATP production occurring via oxidative phosphorylation [37]. However, no similar study has yet examined whether seed deterioration also affects ATP production through sugar metabolism processes.

Spermidine (Spd) is a type of polyamine (PA). In plants, PAs are involved in many physiological processes, including cell division, plant development and differentiation in response to abiotic and biotic stresses, and seed germination [29,38,39,40]. Multiple studies have shown that Spd effectively improves seed germination ability under stress [29,39,41,42]. For example, Spd enhanced starch decomposition and improved germination of wheat seeds under cold stress [41]. Importantly, soaking seeds with Spd significantly improved seed germination performance under drought and osmotic stress, upregulated the activity of α-and β-amylase, decreased starch content, and increased reduced sugar and glucose content [29,43,44]. Adding covalently bound polyamines into the mitochondrial membranes of wheat germ maintained mitochondrial conformation and enhanced wheat tolerance to drought stress [43]. Although seed aging is not a process of stress, many physiological changes are similar to stress; thus, it can be considered a type of time-dependent stress. It was found that starch decomposition is blocked during germination of aged seeds [45,46]. A few previous studies have used Spd to improve seed aging vigor, but largely, it has been used to improve the activity of antioxidant enzymes and related gene expression, ability to scavenge reactive oxygen species, and seed vigor [2,18]. Hitherto, only a few studies have assessed the effect of Spd on sugar metabolism during the germination of aged seeds, and even fewer studies have done so in sorghum seeds.

The objectives of this experiment were to (a) verify whether starch decomposition and sugar metabolism were blocked during germination of aged sorghum seeds; (b) investigate whether exogenous Spd can effectively improve germination of aged sorghum seeds; (c) evaluate how Spd affects sorghum germination through energy metabolism pathways.

## 2. Results

### 2.1. Exogenous Spd Improves Germination Ability of Artificially Aged Sorghum Seeds

**Figure 1:**
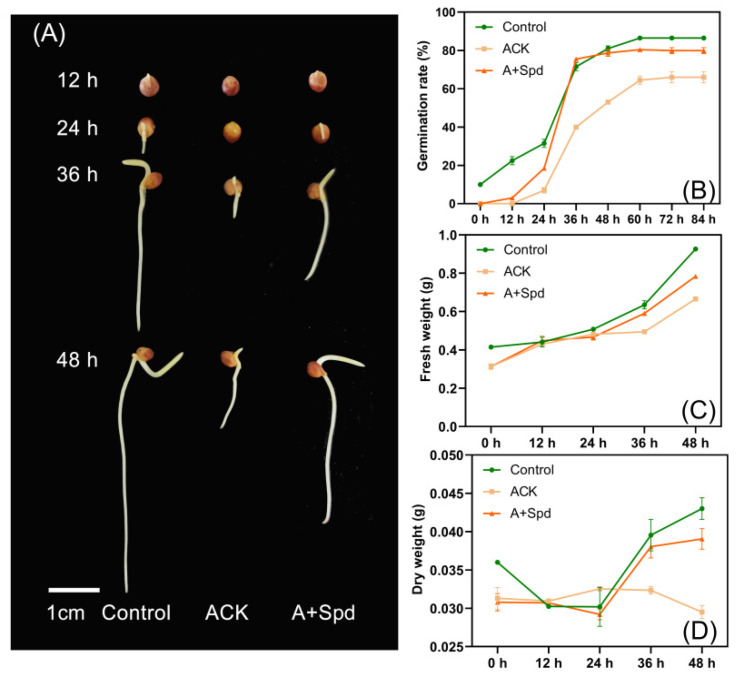
Exogenous Spd enhances germination of aged sorghum seeds. (**A**) germination phenotypes of sorghum seeds for 12–48 h with different treatments (Control, healthy seed; ACK, artificially aged seed; A+Spd, aged seeds with Spd treatment). Scale bar = 1 cm. (**B**) Quantitative analysis of the final germination rate of the different treatments in (**A**). (**C**) Quantitative analysis of fresh weight from 0–48 h of germination of different treatments in (**A**). (**D**) Quantitative analysis of dry weight from 0–48 h of germination of different treatments in (**A**). A 0.05 μM concentration of exogenous Spd was used.

The germination rate of sorghum seeds decreased significantly after artificial aging, and it was 25% lower than that of the Control treatment. From the normally germinated seeds, the root and shoot lengths of the Control treatment were 156.7 mm and 72.4 mm, respectively, and the root and shoot lengths of the aged (ACK) treatment were 96.3 mm and 38.65 mm (Table 1). Although aged seeds were able to grow shoots and roots, the development of root and shoot growth was significantly inhibited (Figure 1). The germination rate of the aged sorghum seed with spermidine (A+Spd) treatment was 27% higher than that of ACK treatment, which reached a significant level. The Spd treatment also improved the germination potential, germination index, and vigor index of aged seeds (Table 1). From the germination rate curves of Figure 1A,B, the germination rate of seeds decreased after aging, and the germination rate reached the maximum value 60 h after germination. Exogenous Spd increased the germination rate of aged seeds, and the germination rate reached the highest level within 36 h of germination. Exogenous Spd increased fresh and dry weights of aged sorghum seeds after germination (Figure 1C,D).

### 2.2. Exogenous Spd Improves Amylase Activity and Starch Conversion Ability during Germination of Artificially Aged Sorghum Seeds

**Figure 2:**
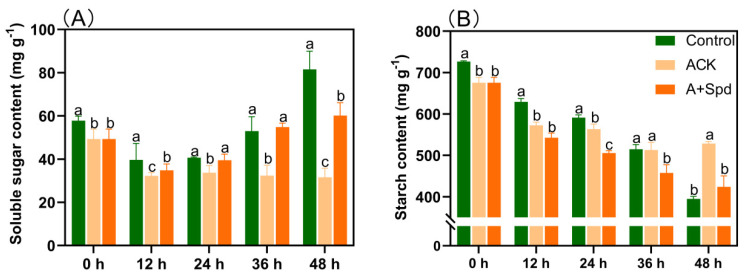
Effects of exogenous Spd on soluble sugar (**A**) and starch (**B**) at the germination stage of aged sorghum seeds. Control−healthy seeds; ACK−artificially aged seeds; A+Spd−aged seeds with Spd treatment. A total of five time points from 0 to 48 h after germination were performed for each treatment, and the different lowercase letters above the bars indicate significantly different levels among the treatments (*p* < 0.05, Lsd). A 0.05 μM concentration of exogenous Spd was used.

**Figure 3:**
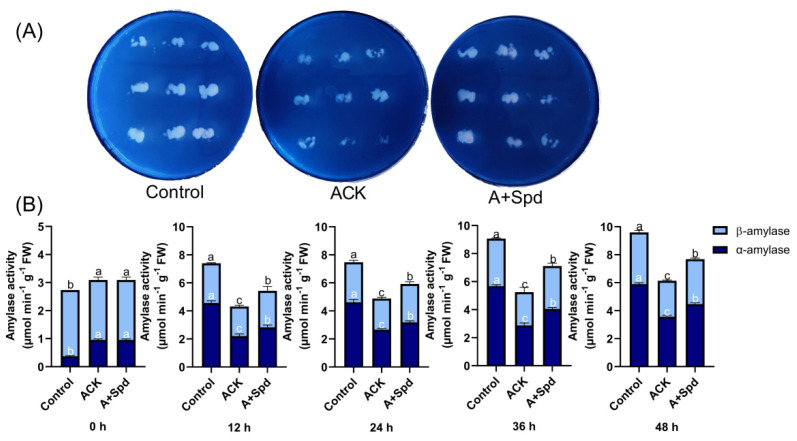
Effects of exogenous Spd on amylase activity in aged seeds. (**A**) the iodine-stained image of starch transformation at 36 h after germination. The size of the white area reflects the transformation ability of seed starch. The larger the white area, the stronger the ability of starch to transform into soluble sugar. (**B**) A quantitative analysis of amylase activity. Control−healthy seeds; ACK−artificially aged seeds; A+Spd−aged seeds with Spd treatment. Different lowercase letters above the bars indicate significantly different levels among the treatments (*p* < 0.05, Lsd). A 0.05 μM concentration of exogenous Spd was used.

As can be seen from Figure 2A, the soluble sugar content increased continuously in the Control treatment during germination. However, the increase in the content of soluble sugar was not obvious in the ACK treatment. It was obvious at each time point that the content of soluble sugar in A+Spd treatment was significantly higher than that in ACK. It is worth noting that the soluble sugar content of A+Spd treatment was not only significantly higher than that of ACK; it was also significantly higher than that of Control treatment at 36 h of germination. From the starch consumption shown in Figure 2B, starch was rapidly decomposed in the Control treatment as germination progressed, while starch decomposition of aged seeds was hindered in the ACK group, and starch decomposition was accelerated in the A+Spd group. To further study the reason why Spd increases soluble sugar content during germination of aged seeds, we determined the activities of several amylolytic enzymes and their ability to break down starch. From the transformation ability of sorghum seed starch in Figure 3A, the white area of starch conversion in the Control treatment was the largest, and its starch conversion ability was the strongest. Aging treatment decreased the ability of sorghum seeds to convert starch, while Spd increased starch conversion. From Figure 3B, the changes in amylase activity had similar effects. At each time point, the amylase activities of the A+Spd treatment, whether α-amylase or β-amylase, were significantly higher than that of ACK treatment. Preliminary conclusions were drawn that Spd could achieve rapid starch conversion by increasing amylase activity.

### 2.3. Exogenous Spd Enhances the Activity of Sucrose Invertase during Germination of Artificially Aged Seeds

**Figure 4:**
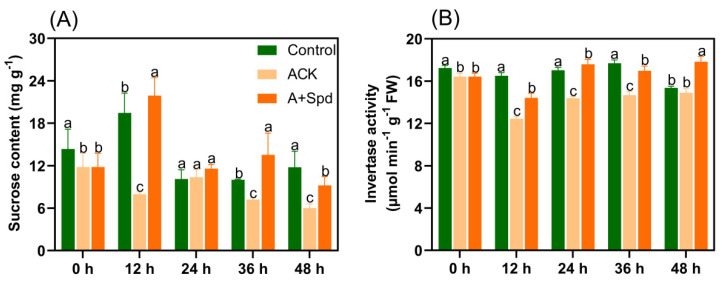
Exogenous Spd enhances sucrose the invertase activity during germination in aged seeds. (**A**) the quantitative analysis of sucrose content. (**B**) The quantitative analysis of sucrose invertase activity. Control−healthy seeds; ACK−artificially aged seeds; A+Spd−aged seeds with Spd treatment. A total of five time points from 0 to 48 h after germination were performed for each treatment. Different lowercase letters above the bars indicate significantly different levels among the treatments (*p* < 0.05, Lsd). A 0.05 μM concentration of exogenous Spd was used.

We found that the Spd treatment increased the concentration of soluble sugars during germination of aged sorghum seeds. To further study the effect of sucrose hydrolysis on the germination ability of sorghum seeds under Spd treatment, we quantitatively analyzed the activity of sucrose invertase during the germination of sorghum seeds, as it is the key enzyme that catalyzes the hydrolysis of sucrose to produce glucose and fructose. The exogenous Spd treatment increased the content of sucrose (Figure 4A). Figure 4B showed that compared with the Control treatment, the invertase activity of aged sorghum seeds was significantly inhibited during the absorption process, while the exogenous Spd treatment improved the sucrose invertase activity in aged seeds.

### 2.4. Exogenous Spd Improves the Internal Energy Supply of Artificially Aged Sorghum Seeds during the Early Stage of Germination by Increasing the Activities of EMP, TCA, and PPP Enzymes

**Figure 5:**
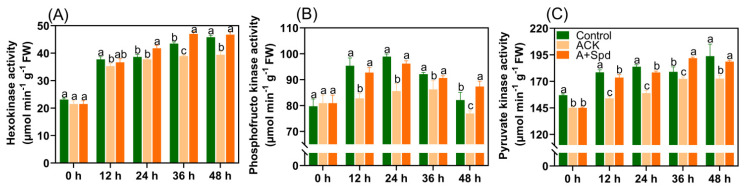
Exogenous Spd increases the activities of key enzymes in EMP during germination of aged seeds. (**A**) The quantitative analysis of Hexokinase activity. (**B**) The quantitative analysis of phosphofructokinase activity. (**C**) The quantitative analysis of pyruvate kinase activity. Control−healthy seeds; ACK−artificially aged seeds; A+Spd−aged seeds with Spd treatment. A total of five time points from 0 to 48 h after germination were performed for each treatment. Different lowercase letters above the bars indicate significant levels of difference among the three treatments (*p* < 0.05, Lsd). A 0.05 μM concentration of exogenous Spd was used.

To explore whether increasing soluble sugar content can be effectively converted into direct energy for germination growth, the key enzyme activities that catalyze the breakdown of glucose to generate ATP were determined. Hexokinase, phosphofructokinase, and pyruvate kinase are three key enzymes of EMP. Here, the activities of these three enzymes were determined in aged seeds 0–48 h after germination. Hexokinase catalyzes the conversion of glucose from a steady state to an active state, and it is the rate-limiting enzyme that catalyzes the first reaction of EMP. In the Control treatment, the activity of Hexokinase gradually increased as the germination time increased, and ACK treatment showed the same trend as the Control treatment, but the activity was significantly lower than that of the Control treatment (Figure 5A). Phosphofructokinase is an important regulator of EMP and gluconeogenesis pathways. In the Control treatment, the phosphofructokinase activity first increased and then decreased as germination time increased, and the aged seeds showed the same trend as the Control treatment, but were significantly lower than the Control treatment (Figure 5B). Pyruvate is the last regulatory enzyme of the glycolytic pathway. Its catalytic reaction ocntrols the exit of the EMP, and its changes are similar to those of Hexokinase (Figure 5C). Finally, we determined the effect of exogenous Spd on the activities of these enzymes. Exogenous Spd treatment activated the activity of Hexokinase in aged seeds. In the time point of 0–48 h of germination, the Hexokinase activity of the A+Spd treatment was significantly higher than that of the ACK treatment. At 36 h of germination, the activity of Hexokinase was not only significantly higher than that of the ACK treatment, but also significantly higher than the Control-treated levels (Figure 5A). There were similar changes in phosphofructokinase and pyruvate kinase activities. Notably, the activity of phosphofructokinase in the A+Spd treatment increased by 12% relative to the ACK treatment at 48 h of germination (Figure 5B). After 36 h of germination, the A+Spd treatment had 1.1-fold higher activity levels of pyruvate kinase than the ACK treatment (Figure 5C).

**Figure 6:**
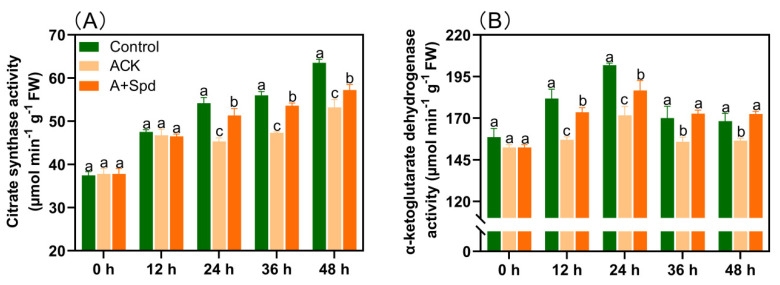
Exogenous Spd increases the activities of key enzymes in the TCA during germination of aged seeds. (**A**) The quantitative analysis of citrate synthase activity. (**B**) Quantitative analysis of α-ketoglutarate dehydrogenase activity. Control−healthy seeds; ACK−artificially aged seeds; A+Spd−aged seeds with Spd treatment. A total of five time points from 0 to 48 h after germination were performed for each treatment. Different lowercase letters above the bars indicate significant levels of difference among the three treatments (*p* < 0.05, Lsd). A 0.05 μM concentration of exogenous Spd was used.

The process of the TCA can generate a large amount of ATP and the precursors required for anabolism. From the quantitative analysis of citrate synthase activity, we can see that the citrate synthase activity in the Control treatment increased by 64.3% from 38.4 μmol min^−1^g^−1^ at the initial germination stage to 63.1 μmol min^−1^g^−1^ with the increase in germination time. In contrast, the activity of this enzyme in the ACK treatment increased by 35% from 37.8 μmol min^−1^g^−1^ to 51.1 μmol min^−1^g^−1^. Then, the activity of citrate synthase after exogenous Spd treatment increased by 52% from 37.8 μmol min^−1^g^−1^ at 0 h of germination to 57.6 μmol min^−1^g^−1^ at 48 h of germination (Figure 6A). Exogenous Spd can significantly improve the activity of citrate synthase. In Figure 6B, the activity of α-ketoglutarate dehydrogenase in the Control treatment increased first and then decreased with the increase in germination time. Although the trend of the ACK treatment was the same, its enzyme activity was significantly lower than that of the Control treatment. The activity of α-ketoglutarate dehydrogenase was significantly increased at each time point after Spd treatment.

**Figure 7:**
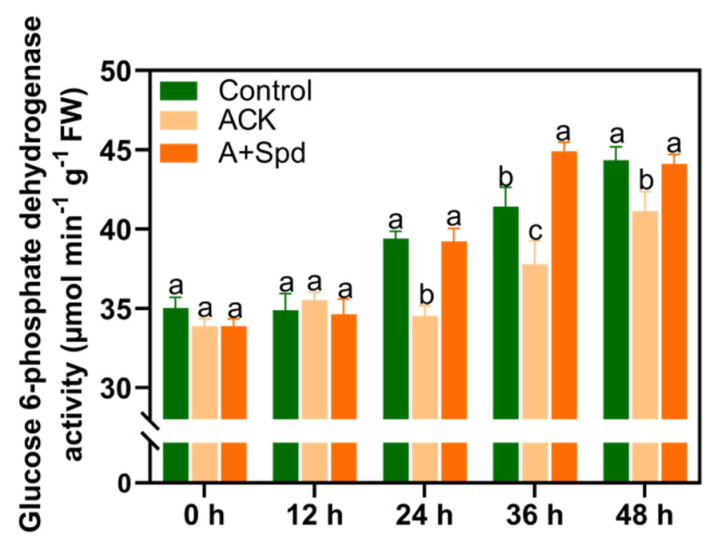
Exogenous Spd enhances the activities of key enzymes in the PPP during germination of aged seeds. Control−healthy seeds; ACK−artificially aged seeds; A+Spd−aged seeds with Spd treatment. A total of five time points from 0 to 48 h after germination were performed for each treatment. Different lowercase letters above the bars indicate significant levels of difference among the three treatments (*p* < 0.05, Lsd). A 0.05 μM concentration of exogenous Spd was used.

The pentose phosphate pathway is carried out in cells, and its function is not to supply energy, but to provide reducing power for intracellular synthesis, and to provide raw materials for the synthesis of intracellular nucleic acid and other substances. Glucose 6-phosphate dehydrogenase is the key enzyme in this pathway. Figure 7 indicates that the activity of glucose-6-phosphate dehydrogenase in Control treatment increased with the germination of seeds. Aging treatment significantly decreased the activity of glucose-6-phosphate dehydrogenase during seed germination. Exogenous Spd significantly increased the activity of glucose-6-phosphate dehydrogenase during germination of aged seeds. Especially at 36 h of germination, the activity of this enzyme was 45.5 μmol min^−1^g^−1^, which was significantly higher than that of the Control treatment at 41.2 μmol min^−1^g^−1^.

**Figure 8:**
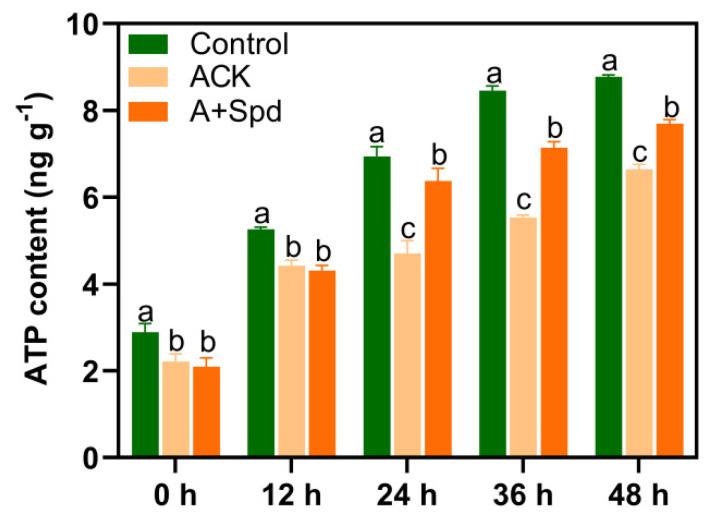
Effects of exogenous Spd on ATP content of aged seeds during germination. Control−healthy seeds; ACK−artificially aged seeds; A+Spd−aged seeds with Spd treatment. A total of five time points from 0 to 48 h after germination were performed for each treatment. Different lowercase letters above the bars indicate significant levels of difference among the three treatments (*p* < 0.05, Lsd). A 0.05 μM concentration of exogenous Spd was used.

ATP is an important molecule involved in energy metabolism, which is required for seed germination and growth. As shown in Figure 8, the ATP content in Control treatment increased with the germination of seeds from 2.9 ng g^−1^ at 0 h to 8.8 ng g^−1^ at 48 h, which increased by 2.03 times. The artificial aging treatment significantly inhibited the production of ATP during seed germination, and the ATP content in ACK treatment increased from 2.3 ng g^−1^ at 0 h of germination to 6.7 ng g^−1^ at 48 h of germination, which increased by 1.91 times. Spd significantly increased the content of ATP in seeds. At 24 h of germination compared with the ACK treatment, it increased by 47%. These results suggest that exogenous Spd positively regulates the conversion of glucose to direct energy substances during germination of aged seeds.

### 2.5. Improvement and Enhancement of Mitochondria and Respiration Rate in Germinating Artificially Aged Seeds by Spd

**Figure 9:**
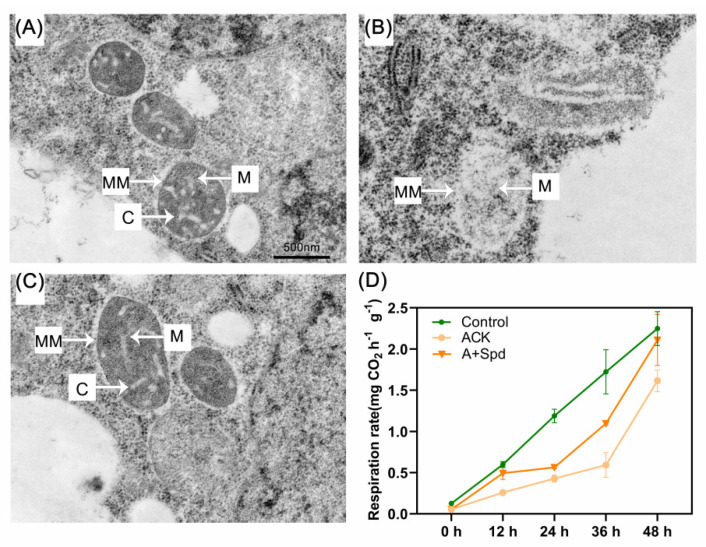
Effects of exogenous Spd on mitochondrial structure and respiration rate during germination in aged embryos. (**A**–**C**) Electron microscope sections of mitochondria in embryos germinated at 36 h under different treatments. (**A**) Control−healthy seeds; (**B**) ACK−artificially aged seeds; (**C**) A+Spd−aged seeds with Spd treatment. Bar = 500 nm, C = cristae, M = mitochondria, MM = mitochondrial membrane. (**D**) Respiration rate analysis of sorghum seeds. Control−healthy seeds; ACK−artificially aged seeds; A+Spd−aged seeds with Spd treatment. The respiration rate of each treatment was measured for seeds at five time points from 0 to 48 h after germination. A 0.05 μM concentration of exogenous Spd was used.

The mitochondrial membranes in the artificially aged embryos were blurred and unclear, and the cristae were difficult to distinguish, and had a tendency to dissolve (Figure 9B). The mitochondrial membrane and cristae were clearly visible after exogenous Spd treatment (Figure 9C). The respiration rate of aged seeds decreased compared with the Control treatment, and the respiration rate of aged seeds was increased by exogenous Spd treatment. (Figure 9D). This result is related to the protection of mitochondrial structure in aging seeds by exogenous Spd treatment.

### 2.6. Exogenous Spd Increases Transcript Levels of Genes Related to Amylase and Sugar Metabolism in Artificially Aged Sorghum Seeds

**Figure 10:**
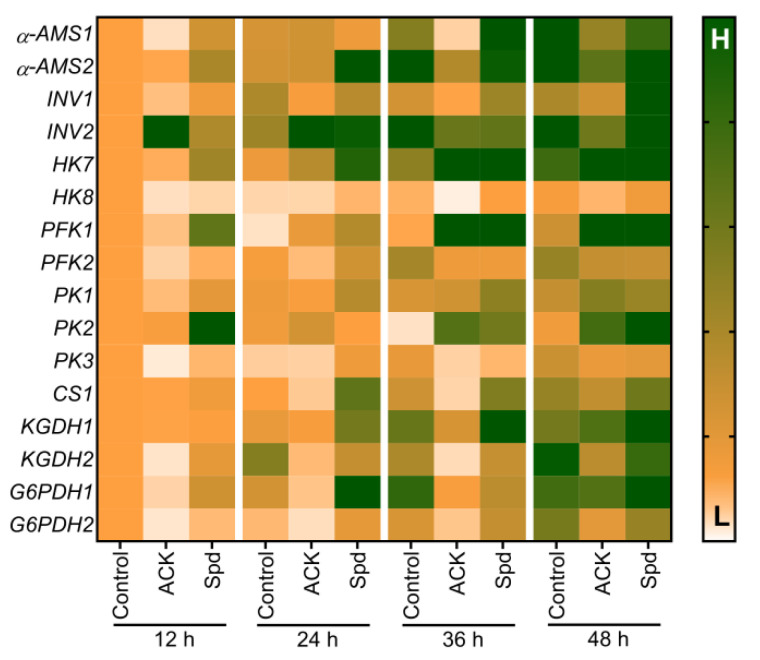
Exogenous Spd treatment increases the transcript levels of genes related to starch and sugar metabolism in aged sorghum seeds during germination. Control−healthy seeds; ACK−artificially aged seeds; A+Spd−aged seeds with Spd treatment. A total of four time points from 12 to 48 h after germination were performed for each treatment. α-*AMS* encodes α-amylase. *INV* encodes sucrose invertase. *HK*, *PFK* and *PK* encode hexokinase, phosphofructokinase, and pyruvate kinase, respectively. *CS* and *KGDH* encode Citrate synthase and α-ketoglutarate dehydrogenase, respectively. *G6PDH* encodes glucose-6-phosphate dehydrogenase. This heatmap was drawn by GraphPad prism software, and gene expression levels ranging from low (L) to high (H) are the smallest and largest in the entire database. A 0.05 μM concentration of exogenous Spd was used.

By studying the expression levels of key genes in starch and sugar metabolism, we further analyzed the role of Spd in improving starch conversion and energy supply during the germination process of aged sorghum seeds. The results of qRT-PCR analysis showed that the artificial aging treatment significantly reduced the transcriptional expression of α-amylase genes (*α-AMS1* and *α-AMS2*), and significantly inhibited the transcription level of the sucrose invertase gene *INV1*. ACK treatment also downregulated the transcriptional levels of *HK8*, *PFK2 PK3*, *CS1*, *KGDH1*, *KGDH2*, *G6PDH1*, and *G6PDH2* of sorghum seeds at 12–48 h of the germination time. It was worth noting that the effect of Spd application on sugar metabolism-related gene expression was opposite to that of ACK treatment. The transcriptional levels of *α-AMS1*, *α-AMS2*, *INV1*, *HK8*, *PFK2*, *PK3*, *CS1*, *KGDH1*, *KGDH2*, *G6PDH1*, and *G6PDH2* were increased in exogenous Spd treatment seeds compared with seeds without Spd treatment (Figure 10). This result is consistent with the effect of Spd on the activities of enzymes related to starch decomposition and sugar metabolism in aged sorghum seeds.

## 3. Discussion

The survival of species and crop yields are largely determined by the quality of seeds. However, seed aging greatly affects their viability and results in germination failure [2]. In this study, we noticed that the vigor and germination capacity of sorghum seeds were significantly inhibited by aging. Importantly, the germination status of aging seeds was significantly improved after exogenous Spd treatment, which was reflected by the significant improvement in rate, potential, and index of sorghum seed germination. Further, Spd treatment boosted the vigor index, as well as the root and shoot length of aging seeds, indicating that exogenous Spd could promote sorghum germination through some metabolic processes.

The energy supply in the seed germination stage comes from the decomposition of storage substances in the seeds, which is starch in the case of sorghum grains. Our experiment found that the starch content in aged sorghum seeds was lower than that in normal seeds before germination, suggesting that starch was consumed during the aging process. This result is consistent with the finding of Jiang et al. [47], who discovered that a large amount of stored substances, such as starch, is depleted during seed aging. Comparison of differentially expressed proteins in seeds of different aging degrees and analysis of KEGG (Kyoto Encyclopedia of Genes and Genomes) pathways showed that the downregulated proteins involved in seed aging were mainly involved in starch and sucrose metabolism [10,48]. Thus, the aging of seeds leads to the gradual decomposition of storage substances and inflicts damage on the metabolism and energy supply system. In turn, the deterioration of the seed negatively affects the energy supply during the germination process, and hence, aging seeds do not germinate normally. In our study, the starch content of normal seeds decreased rapidly after germination, while the starch content of aged seeds decreased at a significantly slower rate than that of normal seeds, resulting in lower soluble sugar content in aged compared to normal seeds, thus affecting the energy metabolism process. These results also suggested that seed aging may affect seeds’ energy supply after germination. In this experiment, exogenous Spd treatment could improve the ability of starch conversion into soluble sugar during germination. This observation concurs with previous studies that have reported that α-amylase/β-amylase activity, fructose and glucose content, and β-amylase gene transcription level in grains were significantly increased after Spd application [29,39]. Thus, the effect of exogenous Spd in promoting germination may be related to relieving starch blockage and releasing the energy required for germination.

The energy supply system plays a vital role in the whole process of seed germination. Transcriptome analysis during seed germination by [26] revealed that the EMP, TCA, and PPP are activated during germination [21,26]. Previous studies on the germination and emergence of aged sunflower and soybean seeds found that the germination rate and seedling establishment were closely related to fatty acid and sugar metabolism. The lack of energy supply caused by the imbalance of fatty acid metabolism and sugar metabolism affected the normal germination of seeds [24,46]. In our experiment, compared with the Control treatment, the activities of enzymes and the expression of genes related to sugar metabolism were inhibited in aged sorghum seeds. Thus, results from cereal as well as oil crops both confirm that the germination ability of aged seeds is affected by the imbalance of sugar metabolism. Hexokinase (HK), phosphofructokinase (PFK) and pyruvate kinase (PK) are key enzymes regulating the EMP. It has been reported that the expression levels of HK, PFK and PK are positively regulated by soluble sugar content in plant tissues [49]. Meanwhile, in the present study, exogenous Spd treatment promoted starch metabolism during the germination of aged sorghum seeds and increased the soluble sugar content, indicating that the increased activities of HK, PFK, and PK in aged sorghum seeds treated with Spd may be the result of increased soluble sugar levels. Kim et al. found that under normal conditions, the OsHXK7, a rice hexokinase, plays a key role in sugar signaling in an EMP-dependent manner [50]. The final product of EMP is pyruvate, which is converted into acetyl-CoA by the pyruvate dehydrogenase complex (PyDC) in the TCA cycle, and becomes the power source for the mitochondrial electron transport chain (ETC) to generate ATP. From the perspective of ATP production capacity in this study, the internal ATP content of aged seeds increased after exogenous Spd treatment, which was closely related to the enhancement of the activities of key enzymes in the EMP and TCA cycles.

Mitochondria are one of the important sites of energy metabolism during seed germination. Analysis of mitochondrial gene dynamic changes confirmed that mitochondrial proteins with multiple functions, such as respiration, metabolism, and transport TCA cycle response proteins, are involved in seed germination [51,52]. In our experiment, transmission electron microscopy revealed that the structure of mitochondria was repaired after exogenous Spd treatment. Therefore, it is likely that the improved germination ability of aged sorghum seeds also depends on Spd regulating the TCA cycle by protecting mitochondria. Furthermore, mitochondria not only play a central role in the two important energy cycles of ETC and TCA, but are also the site of respiration. Consequently, mitochondria provide energy for cell metabolism and transportation through respiration. In agreement, we noticed that the respiration rate of aged seeds increased after germination in response to exogenous Spd application. Therefore, Spd promotes the decomposition and utilization of stored starch and protects mitochondrial structure.

## 4. Materials and Methods

### 4.1. Plant Materials and Growth Condition

The sorghum seeds of Jiza 123 used in this experiment were harvested in 2020 and provided by the Jilin Academy of Agricultural Sciences. All sorghum seeds were stored at 4 °C for later use.

### 4.2. Controlled Deteriorate Treatment Assay (CDT)

The procedures for aging of sorghum seeds were modified from the longevity CDT treatment for soybean seeds described by [24]. The sorghum seeds were completely immersed in 58 °C water for 45 min, and then dried at 25 °C for 2 d. Finally, dried seeds were selected for germination analysis.

### 4.3. Seed Germination Test

Preliminary test results showed that Spd solution with a concentration of 0.05 μM had the best germination effect. The healthy sorghum seeds were cultured under 15 mL of distilled water (Control). Seeds of uniform size were selected, sterilized with 5% sodium hypochlorite solution for 15 min, and the seed surface was rinsed five times with distilled water. The aged sorghum seeds were, respectively, cultured under 15 mL of distilled water (ACK) or 0.05 μM Spd solution (A+Spd). Sorghum seeds were placed in a 15 cm Petri dish containing two layers of filter paper. Each treatment was repeated three times, and each replicate contained 50 seeds. Germination was under complete darkness, at 28 ± 1 °C, and 50% humidity. Seed germination was recorded when radicle length was greater than 2 mm and the germination rate was calculated. In accordance with the experimental requirements, the shoot length, root length, and fresh and dry weight of seedlings were determined. Germinated whole seeds at 0, 12, 24, 36, and 48 h after germination were taken and stored at −80 °C and used for the determination of amylase activity, starch content, soluble sugar content, sugar-metabolizing enzyme activity, RNA extraction, and qRT-PCR.

### 4.4. Germination Characteristics, Morphological Characteristics and Biomass Determination

Gt is the number of germinations on the t day, Dt is the number of germination days, 7 days ≥ t.


(1) $$\mathrm{Germination}\;\mathrm{rate}\;=\frac{\mathrm{the}\;\mathrm{number}\;\mathrm{of}\;\mathrm{normally}\;\mathrm{germinated}\;\mathrm{seeds}\;\mathrm{on}\;\mathrm{the}\;7\mathrm{th}\;\mathrm{day}}{\mathrm{the}\;\mathrm{total}\;\mathrm{number}\;\mathrm{of}\;\mathrm{tested}\;\mathrm{seeds}}\times 100\%\;$$

(2) $$\mathrm{Germination}\;\mathrm{potential}\;=\frac{\mathrm{the}\;\mathrm{number}\;\mathrm{of}\;\mathrm{normally}\;\mathrm{germinated}\;\mathrm{seeds}\;\mathrm{on}\;\mathrm{the}\;4\mathrm{th}\;\mathrm{day}}{\mathrm{the}\;\mathrm{total}\;\mathrm{number}\;\mathrm{of}\;\mathrm{tested}\;\mathrm{seeds}}\times 100\%\;$$

(3) Germination index =∑GtDt

(4) $$\mathrm{Vigor}\;\mathrm{index}\;=\frac{\mathrm{root}\;\mathrm{length}\;\mathrm{of}\;\mathrm{normal}\;\mathrm{germinating}\;\mathrm{seedlings}\;\mathrm{on}\;\mathrm{the}\;7\mathrm{th}\;\mathrm{day}}{\mathrm{number}\;\mathrm{of}\;\mathrm{seeds}\;\mathrm{that}\;\mathrm{germinated}\;\mathrm{normally}\;\mathrm{on}\;\mathrm{the}\;7\mathrm{th}\;\mathrm{day}}\times \;\mathrm{GI}$$

### 4.5. Determination of Soluble Sugar, Sucrose, and Starch Content

First, 0.1 g of whole seeds with roots and shoots was evenly ground with 80% ethanol solution for extraction. Soluble sugar content was determined by using the anthrone method [23]. Sucrose content was measured by using the resorcinol method [53]. Starch was extracted from residues containing extracted soluble sugars using 9.2 moL L^−1^ perchloric acid. The starch content determination is based on the method of Liu et al. [23].

### 4.6. Amylase Activity Assay

First, 1.0 g of germinated sorghum seeds with roots and shoots was weighed and ground into a homogenate. Then, the supernatant obtained following centrifugation at 6000× *g* for 10 min was poured into a 50 mL volumetric flask, and the amylase stock solution was made up to volume with distilled water. Then, 1 mL of these samples was diluted in 50 mL of distilled water as a diluent to determine the (α + β) amylase activities, which were measured using 3,5-dinitrosalicylic acid colorimetry, as described by Hu et al. [18].

### 4.7. Conversion of Starch

Nine sorghum seeds were germinated for 36 h and cut in half with a knife, and then placed cut-side down on the surface of agar medium containing starch. The seeds were kept about 1 cm distance apart on the agar, and the medium was kept at 25 °C for about 30–40 min to hydrolyze the starch. Finally, the seeds were carefully removed with tweezers, and the entire surface of the agar was soaked with dilute I_2_-KI solution. After the blue color appeared, the excess I_2_-KI solution was washed off immediately. The color change range of the place where the seeds were dropped was observed and photographed [54].

### 4.8. Glucose Metabolizing Enzyme Activity Assay

The activities of sucrose invertase, pyruvate kinase, phosphofructokinase, hexokinase, citrate synthase, α-ketoglutarate dehydrogenase, and glucose 6-phosphate dehydrogenase were determined by enzyme-linked immunosorbent assay (ELISA). The kit was provided by Baolai Co., Ltd. (Yancheng, China).

### 4.9. Respiratory Rate Measurement

One gram of fresh seeds germinated at 0, 12, 24, 36, and 48 h was collected to measure the respiration rate using a Carbon dioxide analyzer EGM-5 of Hansha Scientific Instruments Limited (Tai’an, China).

### 4.10. Mitochondrial Transmission Electron Microscope Preparation Steps

Fresh sorghum embryo tissue of 1 mm^3^ in size was collected 36 h after germination, and transferred to an EP tube filled with a new electron microscope fixative for further fixing. Then, the processing method of the sample was referred to Nie et al. [32]. The samples were stained in 2% uranyl acetate saturated alcohol solution for 8 min in the dark. Sections were cut with an ultrathin slicer (LEICA UC7, Leica, Wetzlar, Germany). A transmission electron microscope (HT7700, Hitachi, Tokyo, Japan) was used for observation and image analysis.

### 4.11. Gene Expression Analysis

Total RNA from all samples was extracted using TRIzol reagent (Tiangen) according to the manufacturer’s protocol. Total RNA was treated with DNase I (Takara Bio, Dalian, China) to remove contaminating genomic DNA. RNA purity of each sample was determined using a NanoDrop-2000 spectrophotometer (Thermo Fisher Scientific, Wilmington, DE, USA). Experiments were performed on high-quality samples with a 260/280 ratio of 1.9–2.1 and a 260/230 ratio of ≥2.0. RNA integrity was verified using 2.0% TAE agarose gel electrophoresis. Gene expression was verified using quantitative real-time polymerase chain reaction (qRT-PCR). Each sample was amplified in 3 technical replicates, and all PCR reactions were performed using Bio-Rad CFX96 Real-Time PCR System (Shanhai, China). The 2^−ΔΔCT^ method was used to calculate the relative expression of circular RNA. Primer sequences for qRT-PCR are shown in Supplementary Table S1.

### 4.12. Statistical Analysis

One-way analysis of variance (ANOVA) was used to compare differences between treatments using SPSS18.0 (SPSS Inc., Chicago, IL, USA) with Duncan’s multiple range method, and the significance level was set at *p* < 0.05. The values are expressed as mean ± standard deviation. All images were made with Graphpad Prism 8 software (Graph Pad Software Inc., San Diego, CA, USA).

## 5. Conclusions

In conclusion, our study demonstrated that exogenous Spd could regulate the activity of key enzymes and expression of related genes in the sugar metabolic pathway by promoting starch decomposition, which finally resulted in realizing the mobilization of starch decomposition to ATP production during the germination of aging seeds. In addition, exogenous Spd simultaneously facilitated protection of the mitochondrial structure and improved the respiration rate of aged sorghum seeds. This study provides an effective method for improving germination of aged sorghum seeds by activating seed energy metabolism. Moreover, further studies should be carried out to investigate whether exogenous Spd is also involved in the metabolic process of other major substances (such as protein and fat) in aged seeds for energy supply during germination.

## Figures and Tables

**Table 1:** Exogenous Spd improves germination traits of aged sorghum seeds.

Treatment	Germination Rate	Germination Potential	Germination Index	Vigor Index	Root Length (mm)	Shoot Length (mm)
Control	0.86 ± 0.04 a	0.88 ± 0.03 a	6.82 ± 0.7 a	41.10 ± 0.7 a	156.7 ± 3 a	72.40 ± 4 a
ACK	0.65 ± 0.03 b	0.57 ± 0.05 b	3.66 ± 0.1 b	17.64 ± 0.1 c	96.3 ± 18 c	38.65 ± 7 c
A+Spd	0.83 ± 0.06 a	0.83 ± 0.01 a	6.47 ± 0.2 a	32.14 ± 0.3 b	124.2 ± 7 b	60.80 ± 5 b

These data are obtained from seven days after germination. Control−healthy seeds; ACK−artificially aged seeds; A+Spd−aged seeds with Spd treatment. Different lowercase letters indicate significant difference among treatments (*p* < 0.05, Lsd). A 0.05 μM concentration of exogenous Spd was used.

## Data Availability

All data supporting the findings of this study are available within the paper and within its supplementary data, which are published online.

## Supplementary Materials

The following supporting information can be downloaded at: https://www.mdpi.com/article/10.3390/plants11212853/s1, Table S1: Primers for the qRT-PCR in this study.
