# Supplementary material for: Exogenous Spermidine Promotes Germination of Aged Sorghum Seeds by Mediating Sugar Metabolism
Source: Plants (Basel). 2022 Oct 26;11(21):2853. doi: 10.3390/plants11212853 (PMC9657371; doi:10.3390/plants11212853)
Supplement: Supplementary file 1 [file plants-11-02853-s001.zip › plants-1924069-supplementary.pdf]

Supplemental Table S1. Primers for the qRT-PCR in this study.

Gene name/Gene ID	Forward primer	Reverse primer
actin	CATTCACGAGACTACCTAG	GACGATGTTGCCATATAGA
a-AMS1/8075199	GCAACTGGTGTCCATCAGGA	CTGCGAAGCACCGAATCTTG
a-AMS2/8078919	CCATCCATCTTCTACGATCATT	GTCGATCTCAGCGAGGTA
INV1/8083711	CAGGTGTTCATTAGGGACTTT	AACCCTTGACCAGGACTAT
INV2/8086319	CTGGACTGTGGTGGATTATTT	TTCCCATCCTCCTGTCTATC
HK7/8055699	TCGCATCACAGTCTTCAATC	GACGACCATCTTCCTTGTATC
HK8/8084358	AAGCAGCGTTCTGTTGCTTC	TGCATGTCCAGACCTTGCTT
PFK1/8065903	TCTACAGGATCACCGAGAAG	ATGCGTACTCTCCCTCAA
PFK2/8073561	GATGGTGGAAGAGAGAACATC	CCAGGTACAAAGCCAGTAAA
PK1/8054665	GTGGAGGTTAGGAAGGTACT	CCATCCCTAAATCACCTCTTG
PK2/8084566	CCTACAAGCAGAGTCACATAC	CTTGTGAGGACCAAGATGAG
PK3/8085870	AGCATGCCAAGCGCATTAAG	GCCAGCAATGTTGCACTTGT
CS1/8071625	TTAAGGGTGGTGAACCTTTG	GTTACTGGGAGAGCATCTATTG
KGDH1/8066403	CACTTCGCAAGCAGATACA	CCTAGATGGCCTTCAACATC
KGDH2/8070970	ATACAGGGACTGGTGATGT	CTGTCGCGGTCATTAGAATAG
G6PDH1/110434344	GAGGTAGTCATCGGACAATATG	GCTTTACCAGCCTTGAGAATA
G6PDH2/110436319	ACGCCTCCAACCATCAGAAG	GGCGTTCATATGCCTCAGGA
